# Low-Dose Ketamine Pretreatment Reduces the Incidence and Severity of Myoclonus Induced by Etomidate: A Randomized, Double-Blinded, Controlled Clinical Trial

**DOI:** 10.1097/MD.0000000000002701

**Published:** 2016-02-12

**Authors:** Guan-Nan Wu, Hai-Jun Xu, Fang-Fang Liu, Xian Wu, Hai Zhou

**Affiliations:** From the Department of Anesthesiology, Xuzhou Central Hospital, Xuzhou, China.

## Abstract

Myoclonic movement induced by etomidate is a common but undesirable problem during general anesthesia induction. To investigate the influence of pretreatment with low-dose ketamine on the incidence and severity of myoclonus induced by etomidate, 104 patients were randomized allocated to 1 of 2 equally sized groups (n = 52) to receive either intravenous low-dose ketamine 0.5 mg/kg (group K) or an equal volume of normal saline (group S) 1 minute before induction of anesthesia with 0.3-mg/kg etomidate. The incidence and severity of myoclonus were assessed for 2 minutes after administration of etomidate. Here, we found that the incidence and intensity of myoclonus were both significantly reduced in low-dose ketamine-treated group compared with saline-treated group. The incidence of adverse effects was low and similar between groups. These results demonstrate that intravenous infusion of low-dose ketamine 0.5 mg/kg 1 minute prior to etomidate administration is effective in relieving etomidate-induced myoclonic movements during general anesthesia induction.

## INTRODUCTION

Etomidate is a nonbarbiturate hypnotic drug that provides a rapid onset of action, a stable cardiovascular profile, and minimal respiratory adverse effects. Therefore, it is preferably advocated as an anesthetic induction agent for hemodynamically unstable patients.^1^ However, etomidate has 2 well known and disturbing adverse effects, pain on injection, and myoclonus. Even though pain on injection has been successfully relieved by a new lipid formulation of etomidate (etomidate–lipuro),^2^ myoclonus is still a problem developed in 50% to 80% of nonpremedicated patients.^3^ Myoclonic movements may lead to patient discomfort and be detrimental to those who have only partial cardiovascular reserves. Myoclonus may also increase the risk of prolapse of vitreous content because of high intraocular pressure in patients with open globe injury and be a serious problem in emergency nonfasting conditions.^4^

Although the mechanism of etomidate-induced myoclonus is still not clear, several studies suggest that it may represent a type of seizure.^5–7^ Ketamine, a widely used anesthetic in clinical practice, has shown its therapeutic capacity for control of seizure.^8^ In addition, it has also been demonstrated that ketamine could block the myoclonus induced by opioids administration both in animal^9^ and human.^10^ However, there is no investigation regarding the effectiveness of low-dose ketamine to prevent unwanted myoclonus caused by etomidate.

Therefore, we designed this study to assess the effects of pretreatment with low-dose ketamine on the incidence and severity of myoclonic movements during induction of anesthesia with etomidate.

## MATERIALS AND METHODS

This study was approved by the ethics committee of the Xuzhou Central Hospital. All patients involved have given their written informed consents.

One hundred four patients of both genders with American Society of Anesthesiologists physical status I or II, aged 18 to 65 years, and scheduled for elective surgery under general anesthesia were enrolled in this study from May 1, 2015, to September 30, 2015. Patients with pregnancy, adrenal cortex dysfunction,^11^ neurological diseases, psychiatric disorders, drug allergies, and those who had received analgesics, sedatives, or opioids within the previous 24 hours were excluded.

Patients were randomly assigned to 2 groups of 52 patients each to receive either low-dose ketamine (0.5 mg/kg; group K) or saline (group S). Randomization was achieved by use of computer-generated random number tables. Drugs were prepared in unlabeled 10-mL syringes outside the operating room by an anesthesiologist who was not involved in the induction of anesthesia.

None of the patients received any premedication. After entering the operating room, standard monitors, including electrocardiography, heart rate, noninvasive blood pressure (NIBP), and peripheral capillary oxygen saturation (SpO_2_) were applied. A 20-gauge cannula was inserted into the dorsum of the patient's hand for drug administration and ringer lactate was infused at a rate of 4 to 6 mL/min.

After preoxygenation for 2 minutes, the pretreatment drug, ketamine (# KH140304, Hengrui, China) or saline, was infused over 30 seconds. One minute after infusion, anesthesia was induced with the 0.3-mg/kg etomidate (# 20150409, Enhua Medicine, China) over a period of 1 minute. Patients were observed continuously for myoclonus by an anesthesiologist who was blinded to group allocation. The patients also were not conscious of the pretreatment drugs. Myoclonic movements were defined as involuntary short muscle contractions, which lead to a short, observable movement of body parts. The intensity of myoclonus was clinically graded as 0 = no myoclonus; 1 = mild myoclonus (short movements of a body segment, eg, a finger or wrist only); 2 = moderate myoclonus (mild movements of 2 different muscles, eg, face and leg); and 3 = severe myoclonus (intense clonic movements of 2 or more muscle groups, eg, fast abduction of a limb).^3^

At 2 minutes after administration of etomidate and evaluation of myoclonus, rocuronium (0.6 mg/kg) and fentanyl (3 μg/kg) were given to facilitate tracheal intubation. Maintenance of anesthesia was provided by sevoflurane with 50% oxygen and 50% air. Mechanical ventilation was administered to maintain an end-expiratory carbon dioxide concentration of 35 to 40 mm Hg.

Adverse effects, including nausea, vomiting, sedation, hallucination, and respiratory depression (respiratory rate <10/min) were recorded before administration of etomidate and 2 hours after recovery by another anesthesiologist who was blinded to the groups in order to avoid bias of the investigator who had observed myoclonus. Heart rate, NIBP, and SpO_2_ were recorded every minute during the study period.

The sample size was calculated based on results that were previously published in patients undergoing etomidate induction. We estimated that 46 patients in each group would be required to detect a reduction rate of 0.3 with 90% power at a significance of *P* < 0.05. We factored in a 10% dropout rate and enrolled 52 patients in each group. The results of this study were evaluated using the Graphpad Prism 5.0 (GraphPad Software Inc, San Diego, CA). Continuous variables were described as mean ± standard deviation and differences between groups were analyzed by using unpaired *t* test for normally distributed data. Categorized variables were described as frequency and analyzed by Fisher exact test. Severity of myoclonic movement in 2 groups were presented as frequency and compared by using Fisher exact test. *P* value <0.05 was considered to be statistically significant.

## RESULTS

**FIGURE 1:**
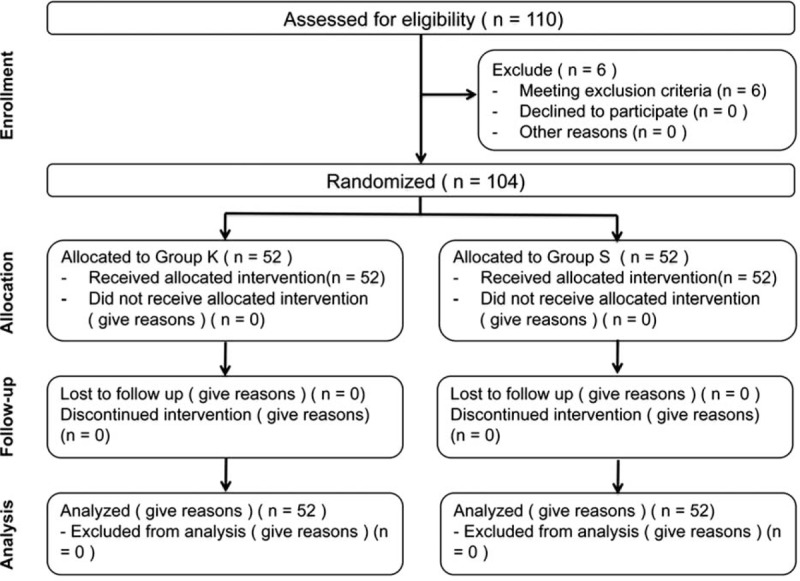
Patient flow (according to the consort chart). Group K = ketamine group, group S = saline group.

In the present study, a total of 104 patients out of 110 consecutive patients met the inclusion criteria and consented for the study. These 104 patients were randomized into 2 groups of 52 each (Figure 1). There were no dropouts or loss to follow-up. The demographic characteristics, with regard to age, gender, body weight, and physical status, were similar between the 2 groups (Table 1).

**TABLE 1:**
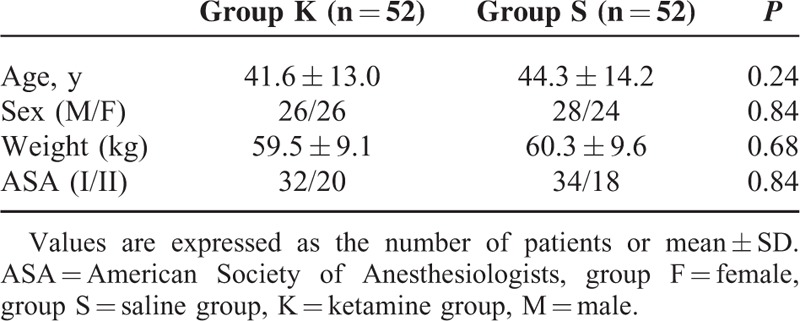
Demographic Data

The incidence of myoclonus after etomidate induction was significantly lower in group K than in group S (23.1% versus 75.0%, *P* *<* 0.001). The severity level of myoclonic movement in group K was also significantly reduced (*P* *<* 0.001). The severe myoclonus was observed for only 1 (1.9%) in group K but 15 (28.8%) in group S (Table 2). The incidences of nausea/vomiting were low and similar in 2 groups, and no patients experienced sedation, hallucination, or respiratory depression (Table 3). The hemodynamic data (NIBP and heart rate) did not differ between 2 groups over the procedure. The SpO_2_ was higher than 97% in all patients. In no case, did the patients experience bradycardia or hypotension during the experimental period.

**TABLE 2:**
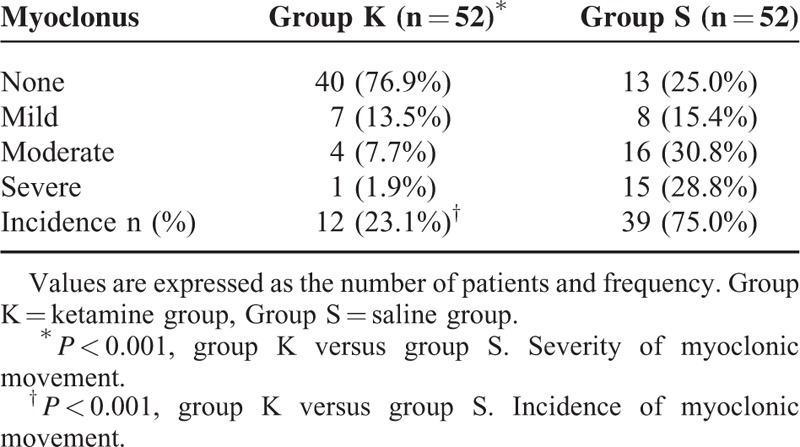
Incidence and Severity of Myoclonus After Etomidate Injection

**TABLE 3:**
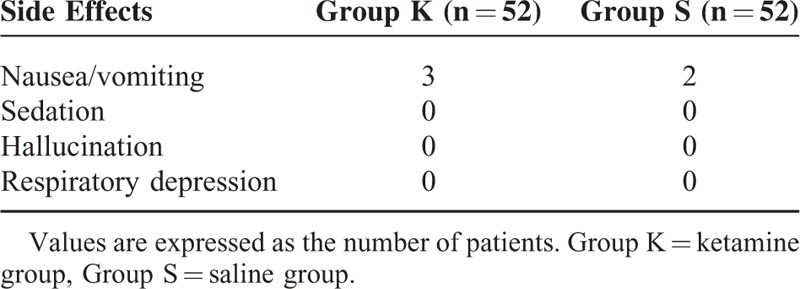
Number of Adverse-Effects

## DISCUSSION

Our study demonstrated that intravenous infusion of 0.5-mg/kg ketamine 1 minute before etomidate administration successfully suppressed etomidate-induced myoclonic movements during the induction of general anesthesia.

Since introduced in 1973, etomidate has been widely used as a general anesthesia induction narcotic in clinical practice. Multiple advantages, such as rapid onset, brevity of action, cardiovascular stability with minimal respiratory adverse effects, and protection of intracranial pressure,^12–15^ make etomidate an ideal agent for rapid general induction, especially for hemodynamically unstable patients. Although the pain on injection has been successfully ameliorated, the etomidate-induced myoclonus remains a common problem, which may lead etomidate an undesirable drug to some anesthesiologists.

Previous studies have introduced a variety of agents that might reduce myoclonus to different extents. Among them, pretreatment with opioids was proven to be the most effective way. High doses of opioids (fentanyl, sufentanil, and remifentanil) effectively reduce myoclonic movements, but at the cost of undesirable adverse adverse effects, including sedation, coughs, apnea, respiratory depression, and chest wall rigidity.^16–18^ Benzodiazepine analog and midazolam have also shown its capacity to reduce myoclonus. The incidence of myoclonus was reduced from 50% in placebo-treated group to 10% in 0.015-mg/kg midazolam-treated group. However, patients suffered a delayed recovery of benzodiazepines.^19^ Muscle relaxant, such as rocuronium (0.06 mg/kg) could reduce the incidence from 63% to 25% by blocking the transmission at neuromuscular junction, but increased the risk of airway obstruction and regurgitation/aspiration.^20^ In addition, lidocaine (20 mg) and thiopental (1.0 mg/kg) could also reduce the incidence of myoclonus with relative low efficacy.^21,22^

Although the neurologic mechanism of etomidate-induced myoclonus is unclear, some studies have suggested that the myoclonic activity might be associated with seizure.^5–7^ Ketamine is a noncompetitive *N*-methyl-d-aspartate receptor antagonist^23^ that might play a role in treating seizure in status epilepticus by blocking *N*-methyl-d-aspartate receptor-mediated glutamatergic neurotransmission.^24,25^ Besides, Kolesnikov et al^9^ reported that ketamine could prevent morphine-induced myoclonus in mice, which is consistent with a clinical report introduced by Forero et al^10^ that low-dose ketamine effectively relieved the painful myoclonus in a patient receiving long-term opioids treatment. In this study, we chose the low-dose ketamine because: low-dose ketamine has shown its capacity to prevent the painful myoclonus^10^; low-dose ketamine administration over 30 seconds could get rid of the psychiatric symptoms induced by large doses (>2 mg/kg, IV) and rapid injection of ketamine (>40 mg/min)^26^; low-dose ketamine premedication has been suggested to be beneficial in improving intubating condition and managing postoperative analgesia.^27,28^ As a result, 0.5-mg/kg ketamine significantly reduced the occurrence of myoclonus from 75.0% to 23.1% and attenuated the severity of myoclonus.

Generally, specific concerns about ketamine use include increased intracranial pressure, increased intraocular pressure, paradoxical myocardial depression, psychodysleptic effects, and accelerated neuronal apoptotic degeneration. However, here, the above adverse effects or related symptoms were not seen, which was in agreement with early studies that low-dose ketamine is safe to use and with low adverse reaction rates.^28,29^

The limitation of our study is that we did not use higher doses of ketamine due to its potential adverse adverse effects. Thereby, further studies are needed to find out whether higher doses of ketamine would exert stronger inhibitory effects without adverse adverse effects and to assess an optimal clinically effective dose of ketamine on etomidate-induced myoclonus.

In conclusion, we observed that pretreatment with low-dose ketamine (0.5 mg/kg) was useful in reducing the incidence and severity of myoclonic movements during induction of general anesthesia with etomidate without any noteworthy adverse effects.
